# Loss of Gα_i_ proteins impairs thymocyte development, disrupts T-cell trafficking, and leads to an expanded population of splenic CD4^+^PD-1^+^CXCR5^+/−^ T-cells

**DOI:** 10.1038/s41598-017-04537-4

**Published:** 2017-06-23

**Authors:** Il-Young Hwang, Kathleen Harrison, Chung Park, John H. Kehrl

**Affiliations:** B Cell Molecular Immunology Section, Laboratory of Immunoregulation, National Institute of Allergy and Infectious Diseases, National Institutes of Health, Bethesda, MD 20892 USA

## Abstract

Thymocyte and T cell trafficking relies on signals initiated by G-protein coupled receptors. To address the importance of the G-proteins Gα_i2_ and Gα_i3_ in thymocyte and T cell function, we developed several mouse models. Gα_i2_ deficiency in hematopoietic progenitors led to a small thymus, a double negative (DN)1/DN2 thymocyte transition block, and an accumulation of mature single positive (SP) thymocytes. Loss at the double positive (DP) stage of thymocyte development caused an increase in mature cells within the thymus. In both models an abnormal distribution of memory and naïve CD4 T cells occurred, and peripheral CD4 and CD8 T cells had reduced chemoattractant responses. The loss of Gα_i3_ had no discernable impact, however the lack of both G-proteins commencing at the DP stage caused a severe T cell phenotype. These mice lacked a thymic medullary region, exhibited thymocyte retention, had a peripheral T cell deficiency, and lacked T cell chemoattractant responses. Yet a noteworthy population of CD4^+^PD-1^+^CXCR5^+/−^ cells resided in the spleen of these mice likely due to a loss of regulatory T cell function. Our results delineate a role for Gα_i2_ in early thymocyte development and for Gα_i2/3_ in multiple aspects of T cell biology.

## Introduction

In thymocytes and peripheral T cells the major functional role ascribed to Gi heterotrimeric G-proteins is to link chemoattractant receptors to downstream effectors that mediate directed cell migration^1^. Numerous G_i_ linked chemoattractant receptors guide the recruitment, trafficking, and the positioning of thymocytes in the thymus and T cells in lymphoid organs^2, 3^. Studies with pertussis toxin revealed an absolute dependence of chemoattractant receptor signaling on Gα_i_ subunits exchanging GTP for GDP^4, 5^. Pertussis toxin ADP ribosylates a cysteine residue near the c-terminus of Gα_i_ proteins preventing chemoattractant receptors from triggering nucleotide exchange. Transgenically expressing the S1 subunit of pertussis toxin using the proximal *lck* promoter led to a severe thymocyte egress defect and a near complete loss of peripheral CD4 and CD8 T cells^6, 7^. However, caveats are needed when interpreting data from experiments employing pertussis toxin. First, pertussis toxin has additional protein targets in cells, whose modification may affect cellular functions and/or homeostasis^8^. Second, pertussis toxin also blocks the exchange activity of Ric-8A, a protein that functions as a Gα_i_, Gα_q_, and Gα_13_ protein chaperone^9, 10^. Third, the B oligomer of pertussis toxin binds glycoconjugate molecules present on mammalian cells and can impact intracellular signaling pathways independent of the enzymatic activity of the S1 subunit. Fourth, pertussis toxin equally affects all Gα_i_ isoforms, thereby only permitting an assessment of their collective inability to undergo receptor initiated nucleotide exchange. The analysis of mice lacking various Gα_i_ subunits avoids some of these problems and provides a complementary approach to pertussis toxin studies.

Both mice and humans express three highly homologous members of the “inhibitory” class of Gα proteins termed Gα_i1_, Gα_i2_, and Gα_i3_^11^. These proteins are encoded by G*nai1*, *Gnai2*, and *Gnai3*, respectively, and each is capable, when GTP bound, to directly inhibit adenylyl cyclase activity. Gene targeting of *Gnai1*, *Gnai2*, and *Gnai3* to create null mutations in mice has revealed redundancy as well as tissue specific functions of the encoded proteins^12–14^. Lymphocytes express little Gα_i1_, and no lymphocyte phenotype has been reported in the *Gnai1* ^−/−^ mice. In contrast, human and mouse lymphocytes strongly express Gα_i2_, and a lesser amount of Gα_i3_. Besides varying expression levels, the proteins localize differently within cells as Gα_i2_ resides predominately on the inner leaflet of the plasma membrane, while Gα_i3_ associates with plasma and intracellular membranes. Mice lacking Gα_i3_ and to a much greater extent Gα_i2_ exhibit immune phenotypes. Three day old neonatal *Gnai3* ^−/−^ mice have reduced numbers of thymocytes and peripheral T cells, however, cell numbers rapidly return to the normal range^15^. In adoptive transfer experiments *Gnai3* ^−/−^ thymic progenitor homed less well to the thymus. Otherwise, no other major defects in lymphocyte function have been reported. In contrast, severe immune defects have been described in the *Gnai2* ^−/−^ mice. They develop a T helper type 1 dominated colitis whose penetrance depends upon the genetic background of the mice^16^. However, C57BL/6 mice kept in a clean mouse facility exhibit little, if any disease. Loss of Gα_i2_ also reduces thymic progenitor homing, and decreased the chemotactic responsiveness of DN1 cells to CXCL12^15, 17^. Double positive (DP) thymocytes more rapidly transit to single positive (SP) cells, which is exaggerated in colitis-prone mice^18, 19^. In the thymus, mature SP thymocytes accumulate, in part secondary to reduced egress^19^ while in the periphery there is an increase in memory T cells^20^. Purified CD4 and CD8 T cells from *Gnai2* ^−/−^ mice respond less well to chemoattractants and, functionally, the *Gnai2* ^−/−^ T cells inefficiently cross endothelial barriers and show reduced migratory rates^21^. They are also poorly retained in lymph nodes following FTY-720 treatment^22^.

Besides underscoring the importance of Gα_i2_ in chemoattractant receptor signaling, the analysis of the *Gnai2* ^−/−^ mice has suggested other functional activities for Gα_i2_ in T cells. For example, compared to wild type T cells, naïve *Gnai2* ^−/−^ CD4 T cells (mixed background) had an enhanced intracellular calcium response, an exaggerated proliferative response, and augmented cytokine production following T cell receptor (TCR) crosslinking^20^. However, treating wild type CD4 T cells with pertussis toxin failed to reproduce these abnormalities. Furthermore, in *Gnai2* ^−/−^ colitis prone mice, regulatory T cells did not normally inhibit effector memory CD4 T cells^23^. While intriguing these studies are complicated by variations in mouse genetic backgrounds, and that thymic T cells development occurs in a globally Gα_i2_ deficient animal or a *Gnai2* ^−/−^ bone marrow reconstituted wild type animal.

To better understand the consequences of loss of Gα_i_ proteins in T cells we have developed several different mouse models to assess the impact of the loss of Gα_i2_ and Gα_i3_ on T cell development, trafficking, and function. These studies confirmed the important of Gα_i2_ in T cell development and Gα_i2_ and Gα_i3_ in both thymocyte and peripheral T cell chemotaxis. They support a role for Gα_i2_ in maintaining naïve T cells and reveal an unexpected phenotype in the double deficient peripheral T cells.

## Results

### Generation of mouse models to assess the impact of the loss of Gα_i_ proteins on thymocyte development

**Figure 1:**
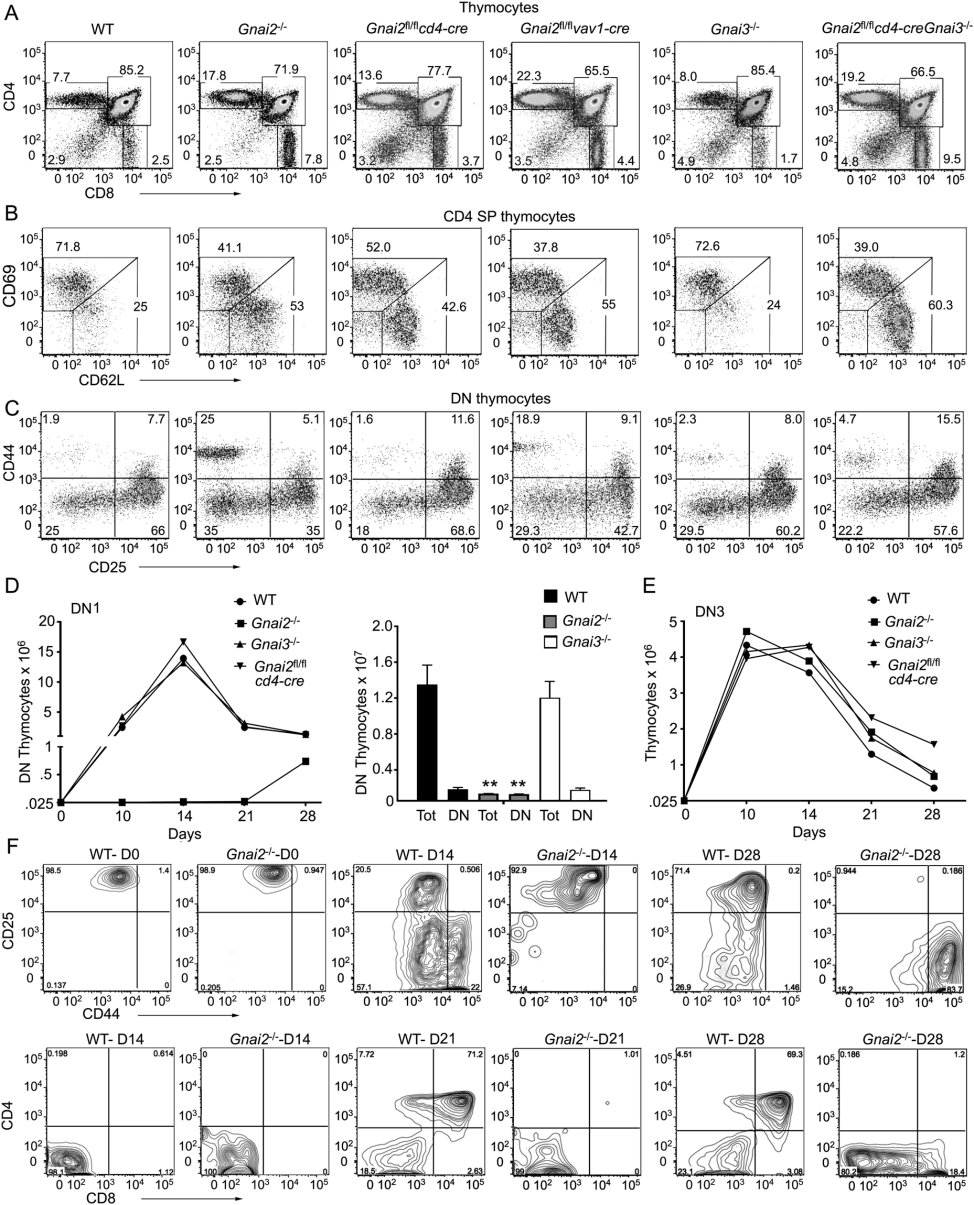
Loss of *Gnai2* inhibits early thymocyte development and causes SP mature thymocytes to accumulate. (**A**) Representative flow cytometry of thymocytes from indicated mice examining CD4 versus CD8 expression. (**B**) Representative flow cytometry of thymocytes from indicated mice gated on CD4 SP thymocytes for their expression of CD62L and CD69. (**C**) Representative flow cytometry of thymocytes from indicated mice gated on DN thymocytes for their expression of CD44 and CD25. (**D**) Growth and differentiation of FACS sorted DN1(Lin^−^CD25^−^ CD44^+^CD117^+^) thymocytes purified from the indicated mice and cultured on OP9-DL1 cells in the presence of IL-7 (1 ng/ml) for 28 days. The numbers of total and DN thymocytes recovered at Day 28 from each of the genotypes is shown to the right. (**E**) Growth of FACS sorted DN3 thymocytes from the indicated mice cultured on OP9-DL1 cells in the presence of IL-7 (1 ng/ml) for 7 days. Experiments were performed a minimum of 3 times. **p < 0.005 (Student’s *t*-test). (**F**) Representative flow cytometry plots from part D comparing WT and *Gnai2* ^−/−^ DN1 thymocytes cultured for various durations on OP9-DL1 cells.

We extensively backcrossed the *Gnai3* ^−/−^ and the *Gnai2* ^fl/fl^ mice onto a C57BL/6 background. Using the *Gnai2* ^fl/fl^ mice, we deleted *Gnai2* in hematopoietic progenitors using *vav1-cre*, and in DP thymocytes using *cd4-cre*. We crossed the *Gnai2* ^fl/fl^ *cd4-cre* mice to *Gnai3* ^−/−^ mice eliminating *Gnai2* and *Gnai3* expression at the DP thymocyte stage. We failed to generate viable *Gnai2* ^fl/fl^ *vav1-cre*/*Gnai3* ^−/−^ mice. Next, we compared the thymocyte profiles of the different mice. Shown are representative FACS profiles of purified thymocytes assessed for CD4 and CD8 expression, and SP CD4 thymocytes for CD62L and CD44 expression, which allows the distinction between immature and mature cells (Fig. 1A and B). The non-conditional loss of Gα_i2_ decreased DP thymocytes and an increased SP CD4 and CD8 cells as previously reported^18, 19^. Also, the % of mature SP CD4 thymocytes was increased. Deleting *Gnai2* using *cd4-cre* led to similar profiles, although the changes were less marked. Conversely, deleting *Gnai2* using *vav1-cre* produced a thymocyte phenotype like that observed in the *Gnai2* ^−/−^ mice. The loss of *Gnai3* had little impact on the thymocyte flow cytometry profiles. The *Gnai2* ^fl/fl^ *cd4-cre*/*Gnai3* ^−/−^ (DKO) mice thymocytes resembled that of the *Gnai2* ^−/−^ and the *Gnai2* ^fl/fl^ *vav1-cre* mice.

We consistently observed a small thymus and reduced thymocyte numbers in the C57BL/6 *Gnai2* ^−/−^ and the *Gnai2* ^fl/fl^ *vav1-cre* mice, but not in the *Gnai2* ^fl/fl^ *cd4-cre* mice suggesting a role for Gα_i2_ in progenitor homing and/or early thymocyte development. To assess early thymocyte development, we examined the expression of CD44 and CD25 on DN thymocytes, which allows the separation of DN thymocytes into 4 consecutive developmental stages termed DN1-DN4^24^. Both the *Gnai2* ^−/−^ and the *Gnai2* ^fl/fl^ *vav1-cre* mice had evidence of a DN1 to DN2 transition block (Fig. 1C). When corrected for the number of thymocytes recovered from the wild type and *Gnai2* ^fl/fl^ *vav1-cre* mice, the later had a three-fold excess of DN1 thymocytes (157,000 versus 62,000), yet one-third fewer DN2 thymocytes (83,000 versus 249,000). There also may be a problem in the DN2-DN3 transition as the WT cells expanded 8-fold while the *Gnai2* ^fl/fl^ *vav1-cre* DN2 cells only expanded 4-fold. Despite a predicted homing defect, the absolute number of early thymocyte precursors^25^ (ETPs, Lin^−^CD4^−^CD25^−^CD44^+^CD117^+^) present in the *Gnai2* ^*fl*/*fl*^ *vav1-cre* mice slightly exceeded those in the WT thymus. A breakdown of the DN3 compartment into DN3a and DN3b cells revealed similar percentages in the WT and the mutant mice suggesting no defect in β chain selection (data not shown). When we tested the expansion of DN1 and DN3 cells from the WT, *Gnai2* ^fl/fl^ *cd4-cre*, *Gnai2* ^−/−^, and *Gnai3* ^−/−^ mice in the OP-9 DL1 culture system, we found a severe defect with the *Gnai2* ^−/−^ DN1 cells (Fig. 1D). They expanded poorly and generated few mature cells. In contrast, the *Gnai2* ^−/−^ DN3 thymocytes exhibited no apparent defect (Fig. 1E). Representative flow cytometry patterns of WT and *Gnai2* ^−/−^ DN1 thymocytes cultured for various durations on OP9 DL1 cells are shown (Fig. 1F). Rather displaying the results as dot blots we used contour plots due to the low number of *Gnai2* ^−/−^ cells recovered from the cultures. In contrast to the WT DN1 cells, the *Gnai2* ^−/−^ DN1 cells failed to generate any DP thymocytes. Overall these results are consistent with a role for Gα_i2_ in the DN1/DN2 thymocyte transition.

### Comparison of T cell compartments in *Gnai2*^*fl*/*fl*^*cd4-cre* and the *Gnai2*^*fl*/*fl*^*vav1-cre* mice

**Figure 2:**
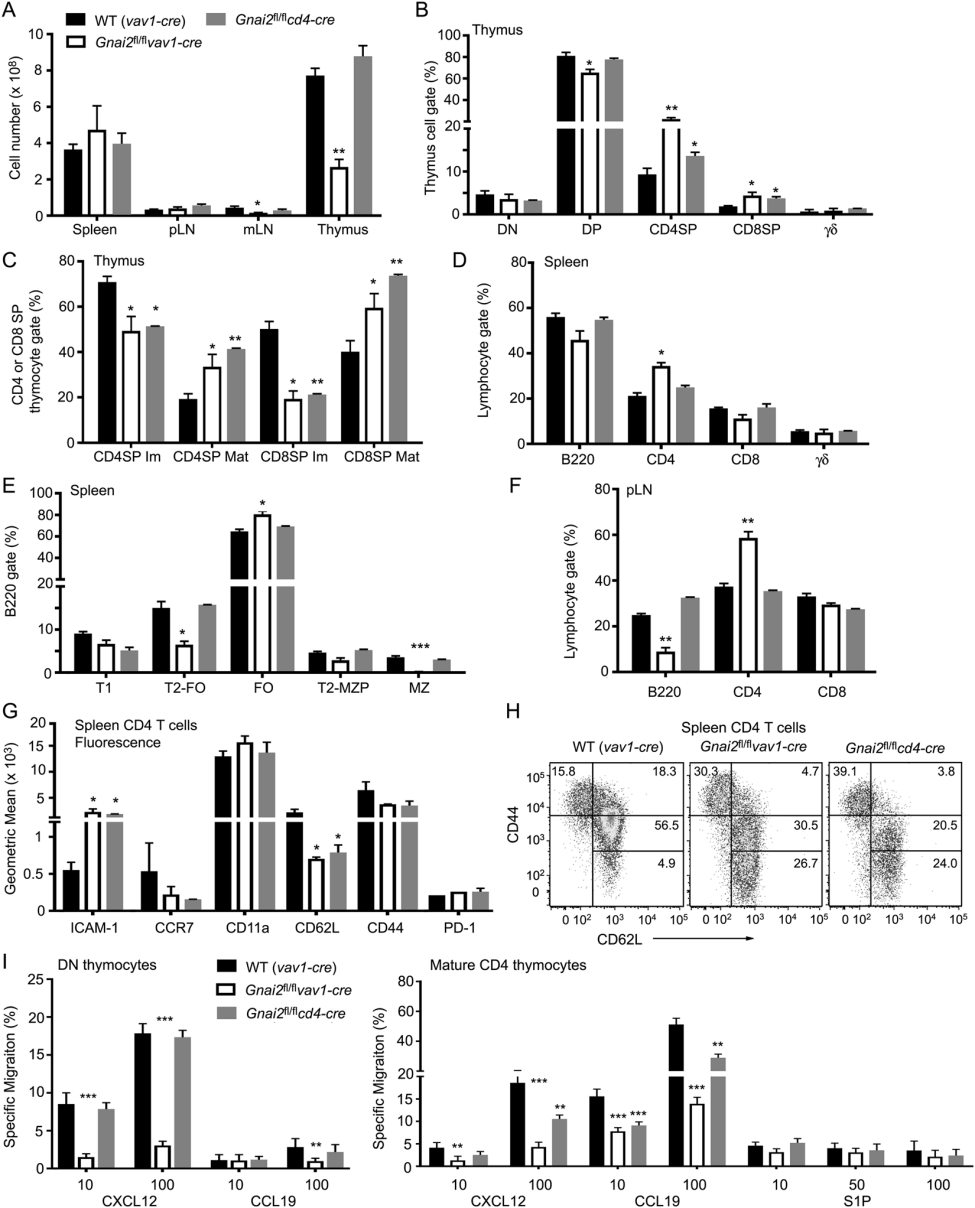
Comparison of deleting *Gnai2* in hematopoietic progenitors versus DP thymocytes. (**A**) Lymphoid organ cell numbers from the indicated mice. (**B**) Flow cytometry results gating on thymocytes to determine the % of DN, DP, SP, and γδ thymocytes. (**C**) Flow cytometry results gating on SP thymocytes and examining CD62L versus CD69 expression. (**D**) Flow cytometry results examining the lymphocyte gate from splenocytes purified from the indicated mice to determine the percentage of B220, CD4, CD8, and γδ cells. (**E**) Flow cytometry results examining B cell development in the spleens of the indicated mice based on the expression of B220, CD21, and CD23. (**F**) Flow cytometry results examining the % of B220, CD4, and CD8 cells in peripheral lymph nodes from the indicated mice. (**G**) Flow cytometry results examining the expression of the indicated marked on splenic CD4 T cells from WT, *Gnai2* ^fl/fl^ *vav1-cre*, and *Gnai2* ^*fl*/*fl*^ *cd4-cre* mice. Results are shown as geometric mean from 4 mice of each genotype. (**H**) Enumeration of naïve and memory CD4 T cells from the spleen of the indicated mice based on expression of CD44 and CD62L. Representative flow cytometry pattern are shown. (**I**) CXCL12, CCL19, and S1P migration assays using DN and mature SP CD4 thymocytes prepared from the indicated mice. Percentages of cells responding to the different chemoattractants are shown. *p < 0.05, **p < 0.005 and ***p < 0.0005 (Student’s *t*-test). Experiments were performed a minimum of 3 times.

Despite the reduction in thymocytes in the *Gnai2* ^fl/fl^ *vav1-cre* mice the number of spleen cells and lymph node cells were like controls with the exception a mild reduction in mesenteric lymph node cells (Fig. 2A). Both the *Gnai2* ^fl/fl^ *vav1-cre* mice and the *Gnai2* ^*fl*/*fl*^ *cd4-cre* mice had an increase in the % of SP thymocytes with a mature phenotype bias (Fig. 2B and C). In addition, the *Gnai2* ^*fl*/*fl*^ *vav1-cre* had an increase in the CD4/CD8 ratio not observed in the *Gnai2* ^*fl*/*fl*^ *cd4-cre*. The *Gnai2* ^fl/fl^ *cd4-cre* mice had a normal B cell compartment while, as expected, the *Gnai2* ^fl/fl^ *vav1-cre* mice had a loss of marginal zone B cells and reduced peripheral B cells (Fig. 2D–F). The B cell phenotype observed in the *Gnai2* ^fl/fl^ *vav1-cre* mice is like that observed following a B cell specific deletion of *Gnai2* ^26^.

In both models peripheral T cells had higher ICAM-1 expression and lower CCR7 and CD62L expression (Fig. 2G). These expression changes may arise from the skewing of the memory/naïve CD4 T cell ratio in the mutant mice (Fig. 2H). An increase in memory/naïve CD4 T cell ratio has also been reported in the *Gnai2* ^−/−^ mixed background mice^20^. Also both strains had an increase in CD62L^high^ CD44^very low^ cells. This subset is reported to be enriched for naïve CD4 T cells with stem cell-like properties^27^. We checked the responsiveness of DN thymocytes to CXCL12 and CCL19 and mature SP CD4 thymocytes to the same chemokines and to S1P. The DN thymocytes from the *Gnai2* ^fl/fl^ *vav1-cre* mice migrated poorly to optimal concentrations of CXCL12, while as expected the *Gnai2* ^fl/fl^ *cd4-cre* mice DN thymocytes responded normally. A similar reduction in CXCL12 directed migration occurred when we examined the DN1 subset from the *Gnai2* ^fl/fl^ *vav1-cre* mice (data not shown). The mature SP CD4 cells from both strains responded less well to CXC12 and CCL19 than did the controls although the Gnai2^fl/fl^ *vav1-cre* CD4 T cells were more impaired. Likely persistent Gα_i2_ protein expression in the *Gnai2* ^fl/fl^ *cd4-cre* SP thymocytes explains the difference. Thus, a loss of *Gnai2* in hematopoietic progenitors reproduces many of the phenotypes reported with the *Gnai2* ^−/−^ mice, while a *cd4-cre* mediated deletion predominately affected late thymocyte development and the composition of the peripheral T cell pool.

### Analysis of the T cell compartments in the *Gnai2*^*fl*/*fl*^*cd4-cre*/*Gnai3*^−/−^ (DKO) mice

**Figure 3:**
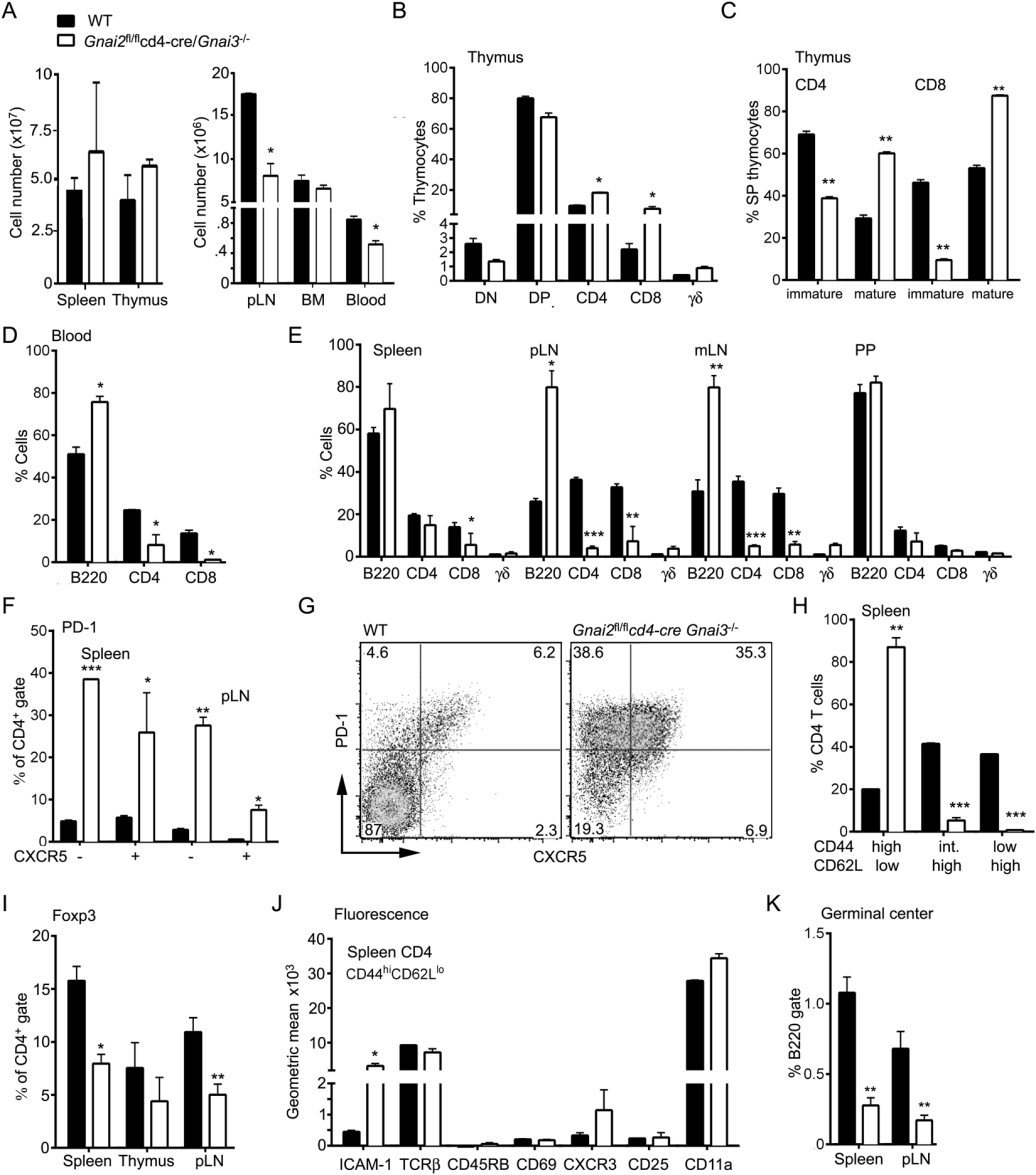
Consequences of deleting *Gnai2* in DP thymocytes of mice that lack *Gnai3* on T cell development and distribution. (**A**) Cell numbers in different lymphoid compartments. Number of cells in the lymphocyte gate. (**B**) Distribution of thymocyte subsets as assessed by flow cytometry. (**C**) Distribution of mature and immature SP CD4 and CD8 thymocytes. (**D**) Distribution of lymphocytes in the blood. (**E**) Distribution of lymphocytes in spleen, peripheral lymph node (pLN), mesenteric lymph nodes (mLN), and Peyer’s patches (PP). Axillary and inguinal lymph node cells were pooled in the pLN analysis. All visible PP were pooled for the analysis. (**F**) PD-1 and CXCR5 expression on CD4 cells from spleen and pLN of indicated mice. (**G**) Flow cytometry pattern of PD-1 versus CXCR5 expression on spleen CD4 cells from WT and DKO mice. (**H**) CD44 and CD62L expression on spleen CD4 T cells. (**I**) Percentage of Foxp3 CD4 T cells in the spleen, thymus, and pLN. (**J**) Comparison of indicated cell surface markers on CD44^hi^CD62L^lo^ splenic CD4 T cells. Data presented as a geometric mean. (**K**) Distribution of germinal center B cells in spleen and pLNs. % of B220 positive cells that express high levels of FAS, GL7, and low levels of CD38. Data represents the analysis of a minimum of 3 mice of each genotype repeated at least three times. *p < 0.05, **p < 0.005 and ***p < 0.0005 (Student’s *t*-test).

Breeding *Gnai2* ^*fl*/*fl*^ *cd4-cre*/*Gnai3* ^+/−^ males and *Gnai2* ^fl/fl^/*Gnai3* ^−/−^ females occasionally generated DKO progeny. The additional loss of *Gnai3* did not affect the numbers of thymocytes or splenocytes when compared to wild type mice, but it caused a reduction in the numbers of blood leukocytes and lymph node cells (Fig. 3A). The thymocyte profile again showed an increase in SP cells with a severe skewing of the mature/immature ratio (Fig. 3B and C). The blood from these mice contained an increased percentage of B220 cells with a reduced percentage of CD4 and CD8 T cells (Fig. 3D). Surprisingly, the spleen contained near normal numbers of CD4 T cells, while lymph nodes had fewer CD4 and CD8 T cells than controls (Fig. 3E). In addition, the number and size of Peyer’s patches were sharply reduced. There was no overt evidence of colitis and splenic B cell development proceeded normally (data not shown). The CD4 T cells in the spleen exhibited an unusual phenotype as nearly 70% expressed PD-1 and half of those co-expressed CXCR5 (Fig. 3F). The residual lymph node CD4 T cells had a similar PD-1^+^CXCR5^+/−^phenotype. There was a near complete loss of naïve CD4 T cells as the splenic and lymph node CD4 T cells lacked CD62L and expressed CD44 (Fig. 3G and H). There was also a significant loss of regulatory T cells in the periphery (Fig. 3I). Further immunophenotyping revealed similar expression levels of CD25 and TCRβ, but increased levels of ICAM-1 and CXCR3 compared to wild type CD4 T cells (Fig. 3J). Despite the CD4 T cell Tfh-like phenotype, the spleen and lymph nodes contained few B cells with a germinal center phenotype (Fig. 3K). Furthermore, while the WT and DKO mice had similar serum levels of IgM, IgG_1_, IgG_2c_, and IgG_3_; the DKO mice had a 50% reduction in serum IgG_2b_ and 25% decrease in IgA (data not shown). Thus, the loss of *Gnai3* and the additional loss of *Gnai2* at the DP stage of thymocyte development led to a reduction in peripheral T cells, but the expansion of an unusual population of CD4 T cells in the spleen.

### Response of DKO thymocytes and splenic T cells to chemokines

**Figure 4:**
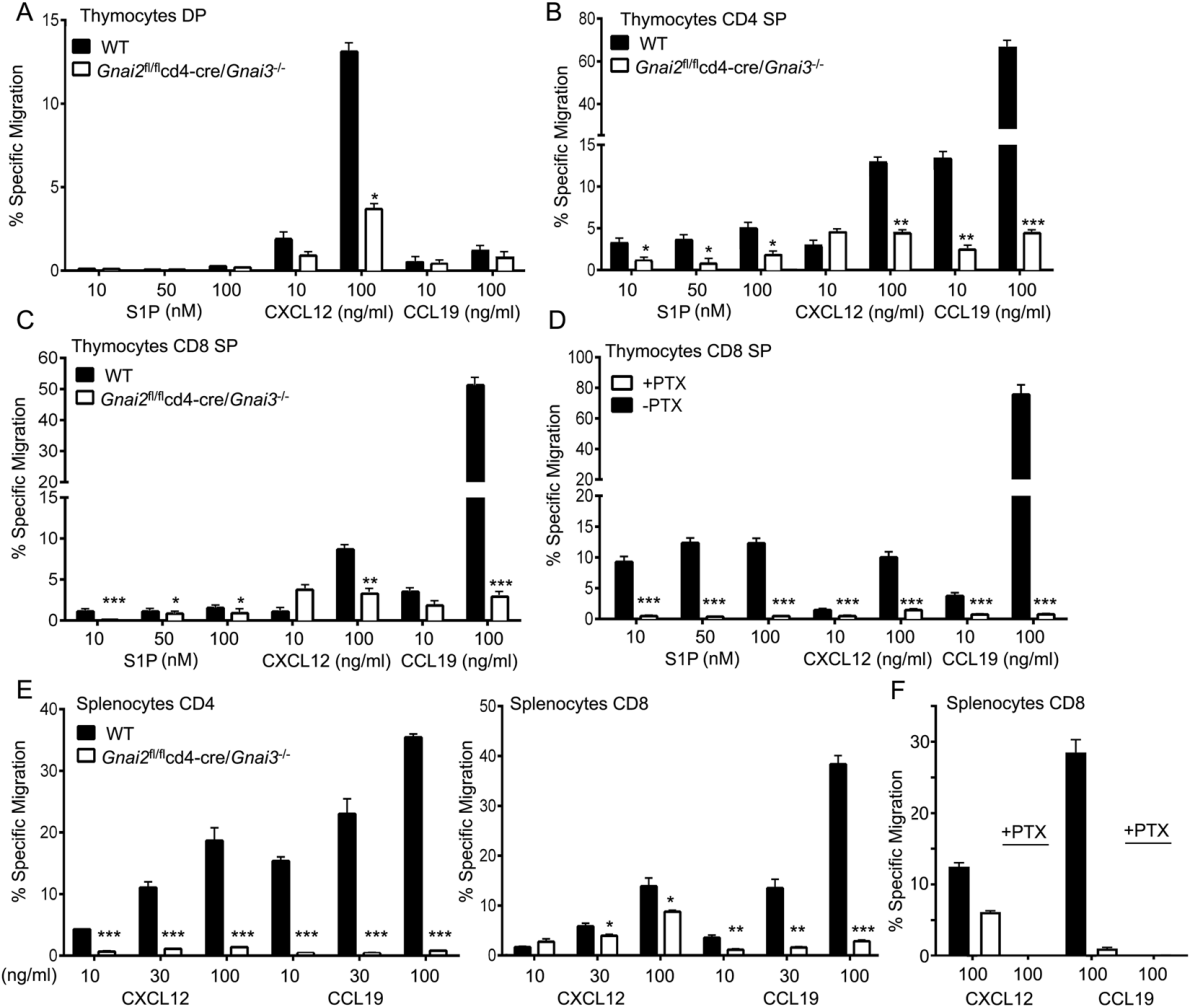
Analysis of migratory capacity of thymocytes and T cells from DKO mice. (**A–C**) Chemotaxis assays with increasing concentrations of S1P, CXCL12, and CCL19 as indicated. Shown are the percentages of DP (**A**), CD4 SP (**B**), and CD8 SP (**C**) thymocytes that specifically migrated. (**D**) Chemotaxis assays with increasing concentrations of S1P, CXCL12, and CCL19 as indicated. Shown are the percentage of CD8 SP thymocytes either pre-treated with pertussis toxin (PTX), or not, that specifically migrated. (**E**) Chemotaxis assays with increasing concentrations of CXCL12 or CCL19 as indicated. Shown are percentages of CD4, CD8, and PTX toxin treated CD8 cells that specifically migrated. Results are 4 mice of each genotype done in duplicate. *p < 0.05, **p < 0.005 and ***p < 0.0005 (Student’s *t*-test).

We found that 13% of the wild type DP thymocytes migrated to CXCL12 while only 4% of the DKO cells responded (Fig. 4A). The SP CD4 and CD8 thymocytes had an approximately 70, 80, and 90% reduction in their responsiveness to optimal concentrations of S1P, CXCL12, and CCL19, respectively (Fig. 4B and C). That some SP DKO thymocytes still specifically migrated to chemoattractant likely reflects persistent Gα_i2_ protein expression as pertussis toxin nearly eliminates all chemokine directed migration (Fig. 4D). The splenic CD4 T cells had a near complete loss in their chemokine responses while the splenic CD8 T cells retained some responsiveness to CXCL12 (Fig. 4E). Consistent with some of the CD8 T cells escaping the CD4-Cre mediated deletion of *Gnai2*, we found that they retained their sensitivity to pertussis toxin treatment (Fig. 4F). As expected splenic B cells from the DKO mice responded normally to CXCL12 and CCL19 (data not shown). These results suggest that nearly all the peripheral CD4 T cells in these mice lack Gα_i2_ and Gα_i3_, while some CD8 T cells have not deleted the floxed *Gnai2*.

### Reconstitution of irradiated wild type or *Rag2*^−/−^ mice with DKO bone marrow

**Figure 5:**
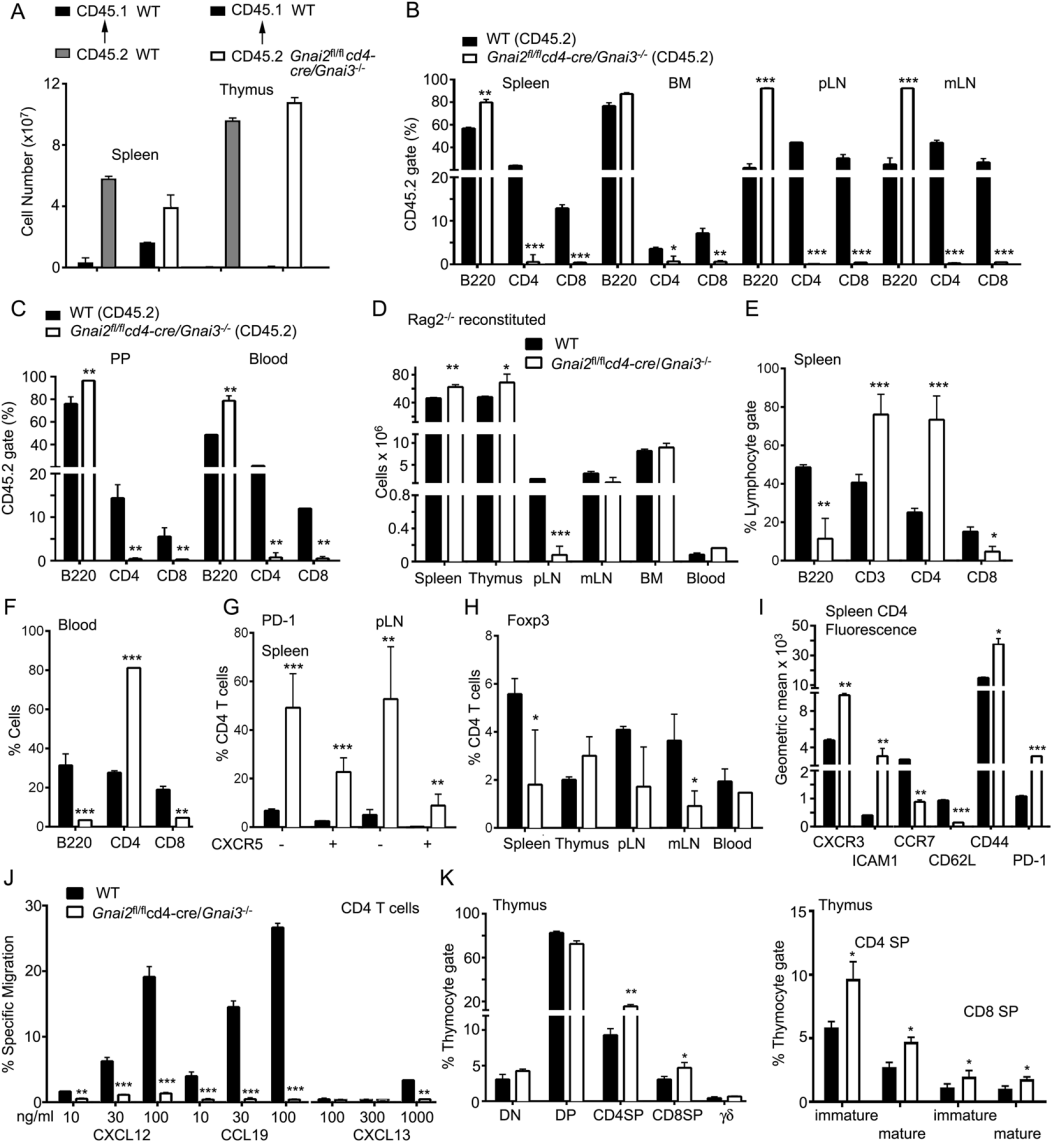
Reconstitution of WT and *Rag2* ^−/−^ mice with DKO bone marrow results in different phenotypes. (**A**) Number of splenocytes and thymocytes recovered from WT reconstituted mice. CD45.1 WT mice were lethally irradiated and reconstituted with bone marrow from CD45.2 WT or CD45.2 DKO mice. Seven weeks later the number of CD45.1 and CD45.2 cells in the spleen and thymus were enumerated. (**B**,**C**) The percentage of different cell types in the lymphocyte gate that also expressed CD45.2. Cells were isolated from the spleen, bone marrow (BM), pLN, and mLN (**B**) and PP and blood (**C**) from the reconstituted mice. (**D**) Number of lymphoid cells recovered from *Rag2* ^−/−^ reconstituted mice. CD45.1 *Rag2* ^−/−^ mice were lethally irradiated and reconstituted with bone marrow from CD45.2 WT or CD45.2 DKO mice. Seven weeks later the number of CD45.1 and CD45.2 cells in various lymphoid organs were enumerated. (**E,F**) Lymphocyte subsets in the spleen (**E**) and blood (**F**) of *Rag2* ^−/−^ reconstituted mice. (**G**) Distribution of PD-1^+^ CXCR5 positive or negative CD4 T cells in the spleen and pLNs of *Rag2* ^−/−^ reconstituted mice. (**H**) Percentage of Foxp3^+^ CD4 T cells in different lymphoid organs and the blood. (**I**) Comparison of indicated cell surface markers on CD4 T cells from *Rag2* ^−/−^ bone marrow reconstituted mice. Data presented as a geometric mean. (**J**) Chemotaxis assays with increasing concentrations of S1P, CXCL12, and CCL19 as indicated. Shown are the percentage of CD4 T cells from *Rag2* ^−/−^ mice reconstituted with the indicated bone marrow that specifically migrated. (**K**) Distribution of thymocyte subsets in *Rag2* ^−/−^ mice with bone marrow from WT or DKO mice. Results are from 8 mice WT or 8 *Rag2* ^−/−^ mice reconstituted either with WT or DKO mice. *p < 0.05, **p < 0.005 and ***p < 0.0005 (Student’s *t*-test).

Because of the difficulties in generating the DKO mice we used bone marrow from these mice or controls to reconstituted irradiated CD45.1 mice. We analyzed the mice 8–12 weeks after reconstitution. We fully reconstituted the thymus however, in the spleen CD45.1 cells persisted, particularly so, in the mice reconstituted with DKO bone marrow (Fig. 5A). The cell populations in the thymus, spleen, lymph nodes, Peyer’s patches, and bone marrow were assessed by gating on CD45.2 positive cells (Fig. 5B). The thymus phenotyping resembled that of the non-reconstituted DKO mice (data not shown), however, the lymphoid organs and the blood had few if any DKO CD4 and CD8 T cells (Fig. 5C). The population of PD-1^+^CXCR5^+/−^ CD4 T cells observed in the non-reconstituted mice had largely disappeared. As regulatory T cells may persist in irradiated hosts^28^, we performed a similar reconstitution experiment using *Rag2* deficient mice as the recipient. In the control and DKO bone marrow reconstituted *Rag2* ^−/−^ mice, we found nearly equivalent cell numbers in the major lymphoid organs and the blood with the exception of peripheral lymph nodes (Fig. 5D). As in the non-reconstituted DKO mice, the DKO bone marrow reconstituted *Rag2* ^−/−^ mice had numerous PD-1^+^CXCR5^+/−^ CD4 T cells in the spleen and the blood (Fig. 5E–G). As previously, the numbers of regulatory T cells derived from the DKO bone marrow were reduced in the periphery (Fig. 5H). Phenotypically the CD4 T cells in the *Rag2* reconstituted mice resembled the CD4 T cells in the non-reconstituted DKO mice with low levels of CCR7 and CD62L, and increased expression of ICAM-1, CXCR3, and CD44 (Fig. 5I). These CD4 T cells were refractory to chemokines (Fig. 5J). The assessment of thymocyte development appeared similar to that noted in the non-reconstituted DKO mice (Fig. 5K). These data suggest that a population of CD4 T cells deficient in Gα_i2/3_ expands in the spleen when regulatory T cells are lacking and those present likely to be dysfunctional.

### Further characterization of the peripheral CD4^+^PD-1^+^CXCR5^+/−^ T cells and memory CD4 T cells from the DKO mice

**Figure 6:**
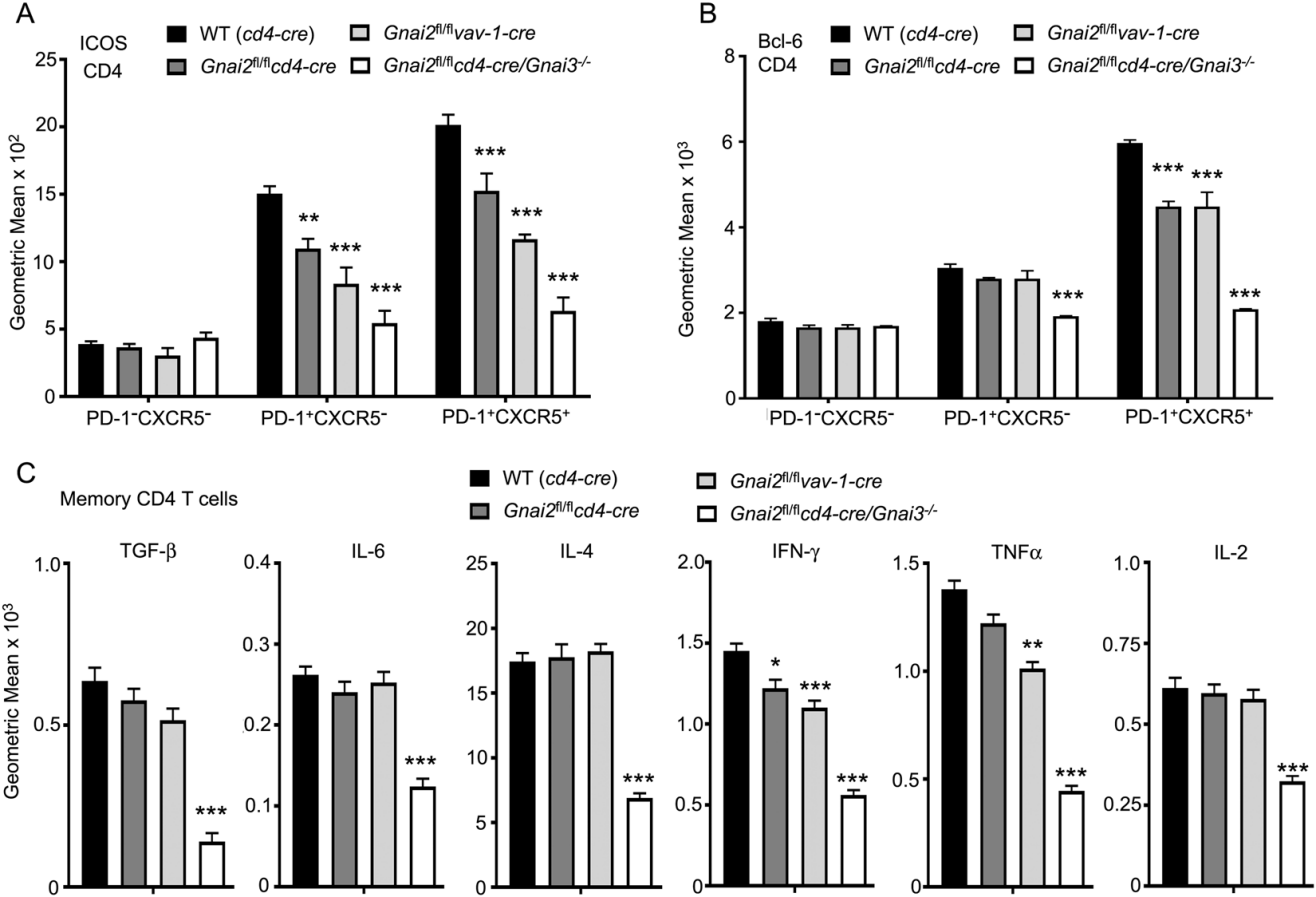
Characterization of CD4 Tfh cells and memory CD4 T cell cytokine production using cells from prepared from different mutant mice. (**A**,**B**) Flow cytometry to examine ICOS (**A**) and intracellular Bcl-6 (**B**) expression by different CD4 T cell subsets. Results are shown as geometric mean from 6 WT, 3 *Gnai2* ^fl/fl^ *vav1-cre*, 6 *Gnai2* ^*fl*/*fl*^ *cd4-cre* and 4 DKO mice. (**C**) Intracellular chemokine levels in memory CD4 T cells isolated from the spleens of WT, *Gnai2* ^fl/fl^ *vav1-cre, Gnai2* ^*fl*/*fl*^ *cd4-cre* and DKO mice. Shown are the geometric means of the immunofluorescence. Purified splenocytes were incubated with Brefeldin A for 10 minutes prior to fixation and immunostaining for CD4, CD44, CD62L, (CD11c, NK1.1, and TCRγδ using the same fluorophore) and the indicated cytokine. CD4 T cells expressing high levels of CD44 and low level CD62L (memory) were assessed for the indicated cytokine expression. Results are from the analysis of 7 WT, 3 *Gnai2* ^fl/fl^ *vav1-cre, 7 Gnai2* ^*fl*/*fl*^ *cd4-cre* and 4 DKO mice. *p < 0.05, **p < 0.005 and ***p < 0.0005 (Student’s *t*-test).

Typical CD4 follicular helper T cells (Tfh) express high levels of PD-1 and CXCR5, and low levels of CCR7^29^ as did the CD4 T cells found in the spleens of the DKO mice. Classic Tfh cells also express high levels of ICOS, the transcription factor Bcl-6, and they produce the cytokine IL-21. In contrast, the DKO CD4 PD-1^+^CXCR5^+^ T cells did not have observable increases in ICOS or Bcl-6. We also noted that the *Gnai2*-deficient Tfh had reduced ICOS and Bcl-6 expression compared to controls (Fig. 6A and B). This suggests some role for Gα_i2_ in generating elevated ICOS expression and a requirement for Gα_i2/3_ for functional Tfh cells.

As noted previously the DKO CD4 T cell compartment essentially lacked naïve CD4 cells as most expressed high levels of CD44 and low levels of CD62L. To provide some assessment of the memory CD4 T cell compartment in the DKO mice, we compared the basal cytokine levels in CD4 CD44^high^CD62L^low^ T cells isolated from WT, *Gnai2* ^*fl*/*fl*^ *cd4-cre*, *Gnai2* ^*fl*/*fl*^ *vav1-cre* or DKO mice. We found that compared to WT cells the DKO CD4 T cells had reduced amounts of all the cytokines tested (Fig. 6C). The *Gnai2* ^*fl*/*fl*^ *cd4-cre*, or *Gnai2* ^*fl*/*fl*^ *vav1-cre* memory CD4 T cells had a small reduction in their basal level of intracellular interferon-γ, but little change in the levels of the other cytokines (Fig. 6C). Thus, CD4 memory cells are likely to be functionally impaired in the DKO mice.

### Loss of the normal thymus and spleen architecture in the DKO mice

**Figure 7:**
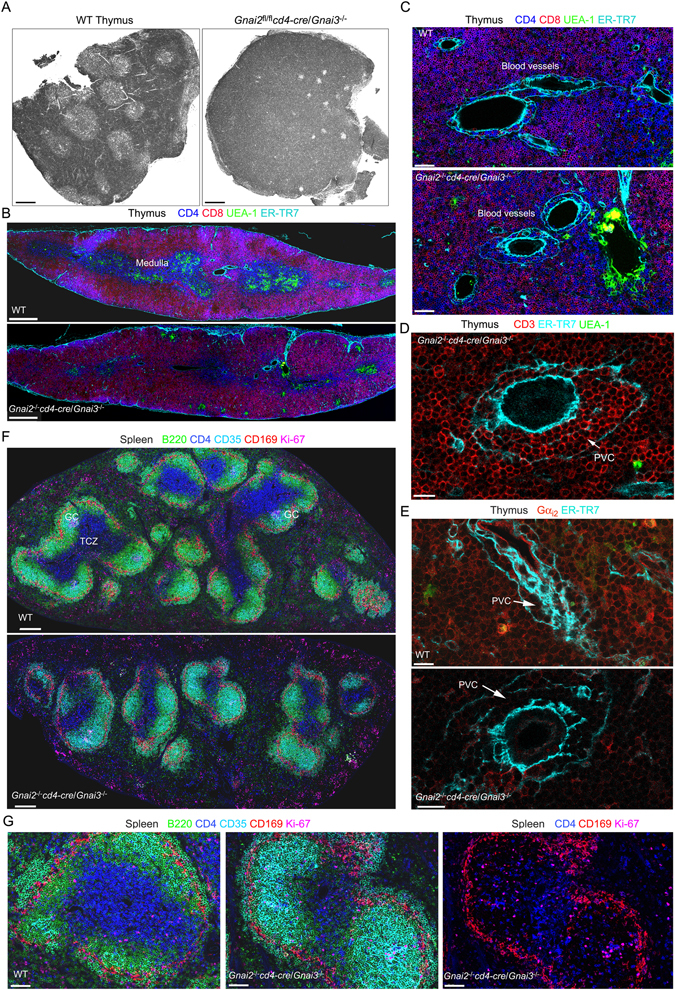
Consequences of deleting *Gnai2* in DP thymocytes of mice that lack *Gnai3* on lymphoid organ architecture. (**A**) Confocal microscopy image collected with a transmission photomultiplier tube of a hematoxylin & eosin stained thymus sections from the indicated mice. Scale bar is 300 μM. (**B**) Confocal microscopy of a thymus section from the indicated mice immunostained for CD4, CD8, UEA-1, and ER-TR7. Acquired images tiled together to span a single thymus lobe. Scale bar are 400 μM. (**C**) Electronically zoomed images from part B focused on a likely site of thymocyte egress. Scale bar is 50 μM. (**D**) Confocal microscopy of a thymus section from a DKO mouse immunostained for CD3, ER-TR7, and UEA-1 focused on a likely egress vessel. The perivascular channel (PVC) is indicated. Scale bar is 15 μM. (**E**) Confocal microscopy of thymus sections from the indicated mice. The sections were immunostained for Gα_i2_ and ER-TR7. The PVC is indicated. Scale bars are 15 μM. (**F**) Tiled confocal images of the spleens of the indicated mice immunostained for B220, CD4, CD35, CD169, and Ki67. The sections are oriented parallel to the short axis of the spleens. Several germinal centers (GC) are evident in the WT spleen. (**G**) Zoomed image of a splenic follicle of the indicated mice from F. The sections were immunostained for B220, CD4, CD35, CD169, and Ki-67. In the far right image the B220 and CD35 signals have been removed. Scale bars are 60 μM.

Hematoxylin and eosin stained thymus sections examined by confocal microscopy using a visible light photomultiplier tube revealed small, fragmented medullary regions in the DKO thymus (Fig. 7A). Thick sagittal sections of the thymus were immunostained with antibodies directed at CD4, CD8, UEA-1, and ER-TR7 (Fig. 7B). UEA-1 serves as a marker for thymic medullary epithelial cells^30^, and ER-TR7 delineates the perivascular space surround egress vessels^31^. Stitched multicolor confocal images showed small, scattered medullary regions in the DKO thymus. Higher magnification showed that thymocytes had accumulated in the perivascular spaces surrounding putative egress blood vessels (Fig. 7C). Immunostaining for CD3 and UEA-1 demonstrated that the perivascular thymocytes had a mature phenotype (Fig. 7D) while immunostaining for Gα_i2_ protein verified the loss of Gα_i2_ in the DKO thymocytes surrounding the egress vessels (Fig. 7E). Analysis of DKO spleen sections for B220, CD4, CD35, CD169, and Ki67 immunoreactivity revealed small T cell zones in the white pulp, while the red pulp contained numerous CD4 cells (Fig. 7F). The splenic red pulp also contained numerous Ki67^+^ cells, some of which expressed CD4. Examining the B cell follicles at higher power demonstrated the presence of numerous CD4 cells and the lack of organized germinal centers (Fig. 7G). Thus, in the DKO mice CD4 T cell populate both splenic white and red pulp, although the usual spleen T cell architecture is severely disrupted.

## Discussion

Several conclusions can be drawn from the previous studies of T cell phenotypes in non-conditional *Gnai2* and *Gnai3* knockout mice^15, 16, 18, 20–22, 32, 33^ and this study of conditionally deleting *Gnai2* in the context of deleting *Gnai3*, or not. First, Gα_i2_ can mediate the essential function of Gα_i_ proteins for thymocyte development when Gα_i3_ is not available, while Gα_i3_ cannot when Gα_i2_ is not available. Second, either the loss of Gα_i2_ in hematopoietic progenitors or the non-conditional loss of Gα_i2_ severely reduces the size of the thymus, interferes with DN thymocyte differentiation, and impairs thymocyte egress. These phenotypes are likely thymocyte intrinsic. The loss of Gα_i2_ at the DP stage is less detrimental resulting in an accumulation of mature SP cells. Third, the conditional loss of Gα_i2_ in either model decreased the responses of peripheral CD4 and CD8 T cells to chemokines, increased the percentage of memory-like CD4 T cells, and enriched for a population of CD4^+^CD62L^high^CD44^verylow^ cells. Fourth, the loss of Gα_i3_ combined with the loss of Gα_i2_ at the DP stage leads to a disorganized thymus with a near absence of a medullary region; decreased DP and increased mature SP thymocytes, and a severe thymocyte egress defect. Fifth, despite the lack of Gα_i2_ and Gα_i3_, mature thymocytes can access the perivascular space of the thymus egress blood vessels. Sixth, despite thymic retention and loss of chemoattractant responsiveness numerous CD4 T cells accumulate in the DKO spleen. Yet when wild type mice are reconstituted with DKO bone marrow these cells are largely eliminated. Finally, functional Tfh and Tregs do not develop in the absence of Gα_i2/3_.

Bone marrow derived T cell progenitors redundantly use the Gα_i_ dependent chemokine receptors CXCR4, CCR7, and CCR9 to enter the neonatal thymus^34^. The lack of Gα_i2/3_ in bone marrow T cell progenitors would likely produce a phenotype like that observed with the triple receptor knockout mice. Unfortunately, we never identified a viable *Gnai2* ^fl/fl^ *vav1-cre*/*Gnai3* ^−/−^ mouse. We did find that 6–12 week old *Gnai2* ^−/−^ and *Gnai2* ^fl/fl^ *vav1-cre* mice had visibly smaller thymuses than the controls. Others have reported fewer thymocytes in *Gnai2* ^−/−^ mice post weaning^17^. Surprisingly, we did not find fewer ETPs in the *Gnai2* ^fl/fl^ *vav1-cre* mice indicating that Gα_i3_ compensated for the loss of Gα_i2_ for entrance into the thymus. The accumulation of DN1 thymocytes in the Gα_i2_ deficient animals suggested a partial block at the DN1/DN2 transition. Upon entering the thymus early progenitors receive signals from the thymic microenvironment that initiate T cell lineage specification and progenitor expansion^35^. The DN1/DN2 transition strongly depends upon Notch1 signaling. Poor access to Notch1 ligands due to the migratory defect present in *Gnai2* ^fl/fl^ *vav1-cre* DN1 thymocytes could account for this observation. However, directly plating Gα_i2_ deficient DN1 cells on OP-9DL1 cells argued against this hypothesis. Further implicating Gα_i2_ in Notch signaling, marginal zone B cell development, which depends upon Notch2, is severely impaired in the *Gnai2* ^fl/fl^ *vav1-cre* mice. Direct plating Gα_i2_ deficient bone marrow progenitors on OP9-DL1 cells does not rescue marginal zone B cell development (I-Y. Hwang, unpublished observation). We are exploring whether the loss of Gα_i2_ impairs the integrin mediated cell-cell interactions needed to trigger Notch receptor processing, or whether the Gα_i2_ deficiency causes a cell intrinsic defect in Notch processing due to impaired Adam10 or γ-secretase activity.

As DN1 thymocytes differentiate to DN2 and DN3 cells they move into the thymic cortex and eventually to the subcapsular zone. Whether this migration is chemokine directed remains controversial^3, 36^. Once they reach the subcapsular zone, the DN3 cells transition to DN4 cells. The conditional deletion of CXCR4 using cre expressed from the proximal *Lck* promoter caused a partial block in the transition of DN3 to DN4 cells^37^. We did not find an obvious DN3-DN4 transition block in the *Gnai2* ^fl/fl^ *vav1-cre* or the Gnai2^−/−^ mice, although the loss of Gα_i2_ did impair the *in vitro* migration of both DN3 and DN4 thymocytes to CXCL12 (3–5 fold decrease). Arguing that Gα_i3_ may compensate for the loss of Gα_i2_ in DN3/4 cells, *Gnai2* ^−/−^ DN3 thymocytes plated on OP9-DL1 stromal cells expanded like wild type DN3 cells.

From the subcapsular zone the DN4 thymocytes migrate into the cortex and toward the medulla^3^. Coincidently they express CD4 and CD8 and transition to the DP stage. CXCR4 levels begin to decrease reducing the cortical retention signal. The deletion of Gα_i2_ prior to entrance into the thymus or under the control of *cd4-cre* had no apparent effect on the appearance of DP cells although DP thymocytes from both strains had reduced response to chemokines. As the thymocyte transit the cortex they undergo positive selection, and subsequently further reduce CXCR4 and increase CCR7 and CCR4 expression. Based on pertussis toxin data entrance into medulla depends upon Gα_i_ signaling although a consensus is lacking on which receptors are required^38^. The non-conditional loss of Gα_i2_ or its loss in hematopoietic progenitors led to a thymus with small, fragmented medullary regions, while the combined loss of Gα_i3_ and Gα_i2_ at the DP stage led to a near absence of thymocytes in the residual medullary regions.

Semi-mature SP thymocytes that successfully undergo negative selection reduce their expression of CD69 and raise their expression of CD62L and the chemoattractant receptor S1PR1, which re-localizes the cells to the corticomedullary junction leading to their Gα_i_ dependent reverse transmigration into the blood via sensing of sphingosine-1 phosphate^39^. The loss of Gα_i2_ interferes with thymocyte access to the medullary region, but it does not prevent the maturation of SP cells. The loss of Gα_i2_ reduces their responsiveness to S1P, which may account for their accumulation in the thymus. The DKO thymocytes have a severely attenuated response to chemokines and S1P, yet they also acquire a mature phenotype. Surprisingly, both the Gα_i2_ deficient and the DKO thymocytes can access the perivenule channels (PVC) surrounding the normal egress blood vessels, although they may have accessed the PVC prior to exhausting their supply of Gα_i2_ protein.

The *cd4-cre* and *vav1-cre Gnai2* ^fl/fl^ mice shared similar peripheral T cell phenotypes. Alteration in thymus egress and responsiveness to chemokines may explain the changes in the CD4 naïve and memory pools we observed. The elevated ICAM-1 expression suggests ongoing immune activation in these mice. However, no overt autoimmunity was noted and the reduction in CD4 ICOS levels does not support ongoing T cell activation. ICOS levels, which normally rise on Tfh cells, did not increase on the Gα_i2_ deficient Tfh-like cells suggesting that CXCR5 signaling supports ICOS expression on Tfh cells. Finally, we found only minimal changes in the basal cytokine levels in naïve or memory CD4 cells that lacked Gα_i2_.

We cannot fully account for the presence of CD4 T cells in the spleen of the DKO mice. Pertussis toxin *lck* transgenic mice apparently lacked these cells^7^. Yet their presence in the periphery of the DKO mice argues that an occasional thymocyte transmigrated into the blood or lymph, despite the loss of Gα_i2/3_ proteins; or some thymocytes escaped from the thymus before cre mediated *Gnai2* deletion, or retained sufficient Gα_i2_ protein to escape. Having left the thymus the newly emigrated CD4 T cells that happened to retain Gα_i2_ expression could access lymph nodes and the splenic white pulp, but as they exhaust their Gα_i2_ supply they would be trapped in lymphoid organs, or relegated to the blood and splenic red pulp. In the DKO mice those CD4 T cells that escaped the thymus expanded, and only a mild reduction in CD4 T cells occurred in the spleen. They evidently proliferated to fill the splenic niche. Many of these splenic CD4 cells expressed high levels of CD44 and PD-1, low levels of CCR7 and CD62L, and some co-expressed CXCR5. Despite this phenotype, they lacked other features of Tfh cells. Regulatory T cells evidently restricted their expansion as the DKO bone marrow reconstituted wild type mice lacked these cells, while they were abundant in DKO bone marrow reconstituted *Rag2* ^−/−^ mice. CD4 T cell made up more than 70% of the lymphocyte gate in the spleen and blood of the reconstituted *Rag2* ^−/−^ mice. The mice exhibited features of a CD4 T cell lymphoproliferative disorder, likely because of the loss of regulatory T cell function.

In conclusion, the homing of thymocyte bone marrow progenitors, thymocyte development, and thymocyte egress partially depends on the availability of Gα_i2_, and completely depends upon Gα_i2_ and Gα_i3_. The loss of Gα_i2_ impairs the expansion of DN1 thymocytes despite the availability of Notch ligands and IL-7. Further investigation of the role of Gα_i_ proteins in Notch signaling in both B and T cell development is warranted. The trafficking of peripheral T cells, their proper localization in lymphoid organs, and their recruitment to inflammatory sites also partially depend upon the availability of Gα_i2_, and completely upon Gα_i2_ and Gα_i3_. If peripheral CD4 T cells lack Gα_i2/3_ they expand and predominately localize in the spleen and blood. Many of these CD4 T cells express high levels of PD-1, ICAM-1, and CD44; and low levels of CD62L, ICOS, and CCR7; and many co-express CXCR5. These cells cannot traffic properly, populating the spleen and accumulating in the blood. Further investigation of mice with more refined deletion of Gα_i_ subunits should provide additional insights to their functional roles in thymocyte development, and mature T cell function.

## Material and Methods

### Mice and bone marrow reconstitutions

C57BL/6, B6.SJL-Ptprc^a^Pepc^b^/BoyJ (CD45.1), C57BL/6 *vav1-cre*, and C57BL/6 *cd4-cre* mice were obtained from Jackson Laboratory. Mice with targeted deletion in *Gnai3* and *Gnai2*, and *Gnai2* ^fl/fl^ mice were kindly provided by Dr. Lutz Birnbaumer (NIEHS, NIH) and backcrossed more than 12 times to C57BL/6 mice^26^. *Gnai2* ^fl/fl^/*cd4-cre* and *Gnai2* ^fl/fl/^ *vav1-cre* mice were obtained by crossing the appropriate cre expressing strain with the *Gnai2* ^fl/fl^ mice and backcrossing to obtain the desired genotype. The *Gnai2* ^*fl*/*fl*^ *cd4-cre*/*Gnai3* ^−/−^ (DKO) mice were generated by breeding a *Gnai2* ^*fl*/*fl*^ *cd4-cre*/*Gnai3* ^+/−^ male mouse versus a *Gnai2* ^fl/fl^/*Gnai3* ^−/−^ female, the only combination that successfully produced viable progeny. For those experiments that directly compared WT and gene targeted mice, littermate controls were used when possible. Otherwise age and sex matched *cd4-cre*, *vav1-cre*, or *Gnai2* ^fl/fl^ mice served as controls. For bone marrow reconstitution, twenty 7 weeks old CD45.1 mice were irradiated twice with 550 rads for total of 1100 rads and received bone marrow from C57BL/6 CD45.2 mice (control) or from the indicated gene targeted CD45.2 mice. The engraftment was monitored by sampling the blood 28 days later. The mice were used 6–8 weeks after reconstitution. All mice were used in this study were 6–14 weeks of age. Mice were housed under specific-pathogen-free conditions. All the animal experiments and protocols used in the study were approved and carried out in accordance with the guidelines of the NIAID Animal Care and Use Committee (ACUC) at the National Institutes of Health.

### Cells

Thymocytes and splenic CD4^+^ T cells were isolated by negative depletion using biotinylated antibodies to B220, CD8, CD11b, Gr-1 (Ly-6C and Ly-6G), NK1.1, TCRγδ, Ter119, CD117 and CD11c and Dynabeads M-280 Streptavidin (Invitrogen). The CD4 T cell purity was routinely greater than 95%. When needed CD4 T cells were cultured in RPMI 1640 containing 10% fetal calf serum (FCS, Gibco), 2 mM L-glutamine, antibiotics (100 IU/mL penicillin and 100 μg/mL streptomycin), 1 mM sodium pyruvate, and 50 µM 2-mercaptoethanol. Cell culture media for sphingosine 1-phosphate (S1P) chemotaxis was same as above except charcoal-dextran filtered fetal calf serum (FCS) was used. On occasion mature thymocytes were isolated from total thymocytes by sorting for cells that expressed CD4, TCRβ, and CD62L, but that lacked CD69 using a FACSAria (BD Biosciences). The sources of the antibodies are listed below.

### OP9-DL1 Culture system

OP9 control and OP9-DL1 cells were obtained from Dr Zúñiga-Pflücker and maintained as recommended previously^40, 41^. The cells were maintained in α-MEM containing 20% fetal calf serum (FCS), antibiotics (100 IU/mL penicillin and 100 μg/mL streptomycin), 1 mM sodium pyruvate, and 50 µM 2-mercaptoethanol. For DN1 (Lin^−^CD44^hi^CD25^−^c-Kit^hi^) and DN3 (Lin^−^CD44^−^CD25^+^) thymocytes were isolated from total thymocytes by sorting using a FACSAria (BD Biosciences). Sorted DN1 (Lin^−^CD44^hi^CD25^−^c-Kit^hi^) and DN3 (Lin^−^CD44^−^CD25^+^) were seeded at 2500 cells/well into 24-well tissue culture plates containing either OP9 control or OP9-DL1 cells to which 1 ng/ml of recombinant mouse IL-7 (Peprotec) was added every two day in α-MEM containing 10% fetal calf serum (FCS), antibiotics (100 IU/mL penicillin and 100 μg/mL streptomycin), 1 mM sodium pyruvate, and 50 µM 2-mercaptoethanol. Stromal cells were changed weekly. The Lin cocktail included anti-CD3 (145-2C11), anti-TCRβ (H57), anti-CD8α (53-6.7), anti-B220 (CD45R), anti CD19 (1D3), anti-CD11b (M1/70), anti-NK1.1 (PK136), anti-GR-1, anti-TCRγδ (GL3), anti-TER 119 and anti-CD11c (HL3).

### Standard flow cytometry

Single cells were re-suspended in phosphate buffered saline (PBS), 2% fetal bovine serum (FBS), and stained with fluorochrome-conjugated or biotinylated antibodies against B220 (RA3-6B2), CD19 (1D3), CD24 (M1/69), CD3 (145-2C11), CD4 (GK1.5 or RM4-5), CD5 (53–7.3), CD8 (53–6.7), CD11c (HL3), CD11b (M1/70), CD117 (2B8), Ter119, CD184 (CXCR4, 2B11), CXCR3 (CXCR3-173), CCR7 (4B12), CXCR5 (2G8), CD11a (M17/4), CD49d (9C10, MFR4.B), CD54 (3E2), CD62L (MEL-16), NK1.1 (PK136), TCRγδ (GL3), TCRβ (H57-597), Ly6G (1A8), Ly6C (AL-21), CD278 (7E.17G9), CD279 (RMP1-30), CD44 (IM7), CD25 (PC61), CD45RB (16A), Qa2 (695H1-9-9), CD69 (H1.2.F3), Bcl-6 (BCL-DWN), CD278 (ICOS), CD45.1 (A20), or CD45.2 (104) (all from eBioscience, Biolegend, or BD Pharmingen). Biotin-labeled antibodies were visualized with fluorochrome-conjugated streptavidin (eBioscience). The intracellular staining of Bcl-6 was performed as described in the manufacturer’s protocol. LIVE/DEAD® Fixable Aqua Dead Cell Stain Kit (ThermoFisher) was used in all experiments to exclude dead cells. Compensation was performed using CompBeads (BD Biosciences) and ArC^TM^ Amine Reactive Compensation Bead individually stained with each fluorochrome. Compensation matrices were calculated with FACSdiva software. Data acquisition was done on FACSCanto II (BD) flow cytometer and analyzed with FlowJo software version 9 (Treestar).

### Intracellular flow cytometry

Brefeldin A (1:1000, eBioscience) was added to block cytokine secretion after harvesting cells from tissue. Labeling of dead cells, fixation, and permeabilization were performed as described in the manufacturer’s protocol. Depending upon the experiment, cells were surface stained with anti-CD4-APC-Cy7 or pacific blue (eBioscience, Biolegend), anti-B220, anti-CD8 and a lineage stain (NK1.1, CD11b, TCRγδ, and CD11c; eBioscience, Biolegend) for 30 minutes at 4 °C and following permeabilization, with anti-IL-2-PE-Cy7 (JES6-5H4; eBioscience), anti-IL-4-PE-Cy7 (11B11; BD Bioscience), anti-IL-6-PE (MP5-20F3; eBioscience), anti-TNFα-PE-Cy7 (TN3-19.12; eBioscience), anti-IFNγ-PE-Cy7 (XMG1.2; Biolegend), anti-TGF-β (R&D) for 30 minutes at room temperature. Cells were finally resuspended in 250 μL 1% BSA/PBS and filtered prior to acquisition on a FACS Canto II flow cytometer (BD Biosciences).

### Chemotaxis assays

Chemotaxis assays were performed using a transwell chamber (Costar) as previously described^42^. The numbers of cells that migrated to the lower well after 2 h or 3 h incubation were counted using a MACSQuant flow cytometer (Miltenyi Biotec). The percent migration was calculated by the numbers of cells of a given subset that migrated into the bottom chamber divided by the total number of cells of that subset in the starting cell suspension, and multiplying the results by 100. D-erythro-sphingosine 1-phosphate was purchased from Avanti Polar Lipids. CCL19 and CXCL12 were purchased (R&D Systems). Fatty acid free bovine serum albumin (FAF-BSA) was purchased (Sigma-Aldrich).

### Confocal microscopy

The thymus and spleen immunofluorescence was performed using a previously described method^43^. Sections were immunostained with the antibodies against CD3, CD4, CD8 (all from eBioscience), CD169 (R&D System), UEA-1 (Vector Laboratories), and ER-TR7 (AbD Serotec) and agitated overnight at 4 °C. Stained thick sections were microscopically analyzed using a Leica SP5 confocal microscope (Leica Microsystem, Inc.) and images were processed with Leica LAS AF software (Leica Microsystem, Inc.) and Imaris v.7.7.1 64x (Bitplane).

### Statistics

*In vivo* results represent samples from 2~5 mice per experimental group. Results represent mean values of at least triplicate samples. Standard errors of the mean (SEM) and *p* values were calculated with *t* test or ANOVA using GraphPad Prism (GraphPad software). *P < 0.05; **P < 0.005; ***and P < 0.0005.

## References

[1] Kehrl JH (2016). The impact of RGS and other G-protein regulatory proteins on Galphai-mediated signaling in immunity. Biochem Pharmacol.

[2] Lian J, Luster AD (2015). Chemokine-guided cell positioning in the lymph node orchestrates the generation of adaptive immune responses. Curr Opin Cell Biol.

[3] Love PE, Bhandoola A (2011). Signal integration and crosstalk during thymocyte migration and emigration. Nat Rev Immunol.

[4] Spangrude GJ, Sacchi F, Hill HR, Van Epps DE, Daynes RA (1985). Inhibition of lymphocyte and neutrophil chemotaxis by pertussis toxin. J Immunol.

[5] Cyster JG, Goodnow CC (1995). Pertussis toxin inhibits migration of B and T lymphocytes into splenic white pulp cords. J Exp Med.

[6] Chaffin KE, Perlmutter RM (1991). A pertussis toxin-sensitive process controls thymocyte emigration. Eur J Immunol.

[7] Chaffin KE (1990). Dissection of thymocyte signaling pathways by *in vivo* expression of pertussis toxin ADP-ribosyltransferase. EMBO J.

[8] Carbonetti NH (2010). Pertussis toxin and adenylate cyclase toxin: key virulence factors of Bordetella pertussis and cell biology tools. Future Microbiol.

[9] Boularan C (2015). B Lymphocyte-Specific Loss of Ric-8A Results in a Galpha Protein Deficit and Severe Humoral Immunodeficiency. J Immunol.

[10] Woodard GE (2010). Ric-8A and Gi alpha recruit LGN, NuMA, and dynein to the cell cortex to help orient the mitotic spindle. Mol Cell Biol.

[11] Hamm HE, Gilchrist A (1996). Heterotrimeric G proteins. Curr Opin Cell Biol.

[12] Jiang M (2002). Mouse gene knockout and knockin strategies in application to alpha subunits of Gi/Go family of G proteins. Methods Enzymol.

[13] Plummer NW (2012). Development of the mammalian axial skeleton requires signaling through the Galpha(i) subfamily of heterotrimeric G proteins. Proc Natl Acad Sci USA.

[14] Offermanns S, Simon MI (1998). Genetic analysis of mammalian G-protein signalling. Oncogene.

[15] Jin Y, Wu MX (2008). Requirement of Galphai in thymic homing and early T cell development. Mol Immunol.

[16] Rudolph U (1995). Ulcerative colitis and adenocarcinoma of the colon in G alpha i2-deficient mice. Nat Genet.

[17] Elgbratt K, Bjursten M, Willen R, Bland PW, Hornquist EH (2007). Aberrant T-cell ontogeny and defective thymocyte and colonic T-cell chemotactic migration in colitis-prone Galphai2-deficient mice. Immunology.

[18] Zhang Y, Finegold MJ, Jin Y, Wu MX (2005). Accelerated transition from the double-positive to single-positive thymocytes in G alpha i2-deficient mice. Int Immunol.

[19] Elgbratt K, Jansson A, Hultgren-Hornquist E (2012). A quantitative study of the mechanisms behind thymic atrophy in Galphai2-deficient mice during colitis development. PLoS One.

[20] Huang TT (2003). TCR-mediated hyper-responsiveness of autoimmune Galphai2(−/−) mice is an intrinsic naive CD4(+) T cell disorder selective for the Galphai2 subunit. Int Immunol.

[21] Hwang IY, Park C, Kehrl JH (2007). Impaired trafficking of Gnai2+/− and Gnai2−/− T lymphocytes: implications for T cell movement within lymph nodes. J Immunol.

[22] Zhi L (2011). FTY720 blocks egress of T cells in part by abrogation of their adhesion on the lymph node sinus. J Immunol.

[23] Gotlind YY, Raghavan S, Bland PW, Hornquist EH (2011). CD4 + FoxP3 + regulatory T cells from Galphai2−/− mice are functionally active *in vitro*, but do not prevent colitis. PLoS One.

[24] Michie AM, Zuniga-Pflucker JC (2002). Regulation of thymocyte differentiation: pre-TCR signals and beta-selection. Semin Immunol.

[25] Tan C (2011). Ten-color flow cytometry reveals distinct patterns of expression of CD124 and CD126 by developing thymocytes. BMC Immunol.

[26] Hwang IY (2013). The loss of Gnai2 and Gnai3 in B cells eliminates B lymphocyte compartments and leads to a hyper-IgM like syndrome. PLoS One.

[27] Zhao C, Marrero I, Narsale A, Moya R, Davies JD (2015). CD4(+) CD44(v.low) cells are unique peripheral precursors that are distinct from recent thymic emigrants and stem cell-like memory cells. Cell Immunol.

[28] Qu Y (2010). Gamma-ray resistance of regulatory CD4 + CD25 + Foxp3+ T cells in mice. Radiat Res.

[29] Crotty S (2014). T follicular helper cell differentiation, function, and roles in disease. Immunity.

[30] Hiramine C, Hojo K, Koseto M, Itoh M (1989). The effect of cyclosporine on murine thymic epithelial cells–an immunohistochemical study. Thymus.

[31] Maeda Y (2014). S1P lyase in thymic perivascular spaces promotes egress of mature thymocytes via up-regulation of S1P receptor 1. Int Immunol.

[32] Wu JY (2005). Impaired TGF-beta responses in peripheral T cells of G alpha i2^−/−^ mice. J Immunol.

[33] Dalwadi H (2003). B cell developmental requirement for the G alpha i2 gene. J Immunol.

[34] Calderon L, Boehm T (2011). Three chemokine receptors cooperatively regulate homing of hematopoietic progenitors to the embryonic mouse thymus. Proc Natl Acad Sci USA.

[35] Shah DK, Zuniga-Pflucker JC (2014). An overview of the intrathymic intricacies of T cell development. J Immunol.

[36] Plotkin J, Prockop SE, Lepique A, Petrie HT (2003). Critical role for CXCR4 signaling in progenitor localization and T cell differentiation in the postnatal thymus. J Immunol.

[37] Trampont PC (2010). CXCR4 acts as a costimulator during thymic beta-selection. Nat Immunol.

[38] Suzuki G (1999). Pertussis toxin-sensitive signal controls the trafficking of thymocytes across the corticomedullary junction in the thymus. J Immunol.

[39] Cyster JG, Schwab SR (2012). Sphingosine-1-phosphate and lymphocyte egress from lymphoid organs. Annu Rev Immunol.

[40] De Smedt M, Hoebeke I, Plum J (2004). Human bone marrow CD34+ progenitor cells mature to T cells on OP9-DL1 stromal cell line without thymus microenvironment. Blood Cells Mol Dis.

[41] Dhanasekaran N, Tsim ST, Dermott JM, Onesime D (1998). Regulation of cell proliferation by G proteins. Oncogene.

[42] Hwang IY, Hwang KS, Park C, Harrison KA, Kehrl JH (2013). Rgs13 constrains early B cell responses and limits germinal center sizes. PLoS One.

[43] Hwang IY (2015). An essential role for RGS protein/Galphai2 interactions in B lymphocyte-directed cell migration and trafficking. J Immunol.

